# *Beet necrotic yellow vein virus *accumulates inside resting spores and zoosporangia of its vector *Polymyxa betae BNYVV infects P. betae*

**DOI:** 10.1186/1743-422X-4-37

**Published:** 2007-04-05

**Authors:** Jeanmarie Verchot Lubicz, Charles M Rush, Mark Payton, Terry Colberg

**Affiliations:** 1 Oklahoma State University, Department of Entomology and Plant Pathology, 127 Noble Research Center, Stillwater, OK 74078, USA; 2 Texas Agricultural Experiment Station, 2301 Experiment Station Road, Bushland, TX 79012, USA; 3 Oklahoma State University, Department of Statistics, Stillwater, OK 74078, USA; 4 Oklahoma State University, Electron and Confocal Microscopy Facility, Stillwater, OK 74078, USA

## Abstract

**Background:**

Plasmodiophorids and chytrids are zoosporic parasites of algae and land plant and are distributed worldwide. There are 35 species belonging to the order *Plasmodiophorales *and three species, *Polymyxa betae*, *P. graminis*, and *Spongospora subterranea*, are plant viral vectors. Plasmodiophorid transmitted viruses are positive strand RNA viruses belonging to five genera. *Beet necrotic yellow vein virus *(BNYVV) and its vector, *P. betae*, are the causal agents for rhizomania.

**Results:**

Evidence of BNYVV replication and movement proteins associating with *P. betae *resting spores was initially obtained using immunofluorescence labeling and well characterized antisera to each of the BNYVV proteins. Root cross sections were further examined using immunogold labeling and electron microscopy. BNYVV proteins translated from each of the four genomic and subgenomic RNAs accumulate inside *P. betae *resting spores and zoospores. Statistical analysis was used to determine if immunolabelling detected viral proteins in specific subcellular domains and at a level greater than in control samples.

**Conclusion:**

Virus-like particles were detected in zoosporangia. Association of BNYVV replication and movement proteins with sporangial and sporogenic stages of *P. betae *suggest that BNYVV resides inside its vector during more than one life cycle stage. These data suggest that *P. betae *might be a host as well as a vector for BNYVV

## Background

There is a group of soilborne plant viruses transmitted by vectors belonging to the Orders *Plasmodiophorales *(*Polymyxa *spp and *Spongospora *spp) and *Chytridales *(*Olpidium spp*). These viruses are positive strand RNA viruses belonging to nine genera. Plant viruses belonging to the genera *Bymo-*, *Beny*-, *Furo*-, *Peclu-*, and *Pomovirus *are vectored by plasmodiophorids. These viruses are internalized by their vector and can remain in the soil for many seasons [1-3].

**Figure 1:**
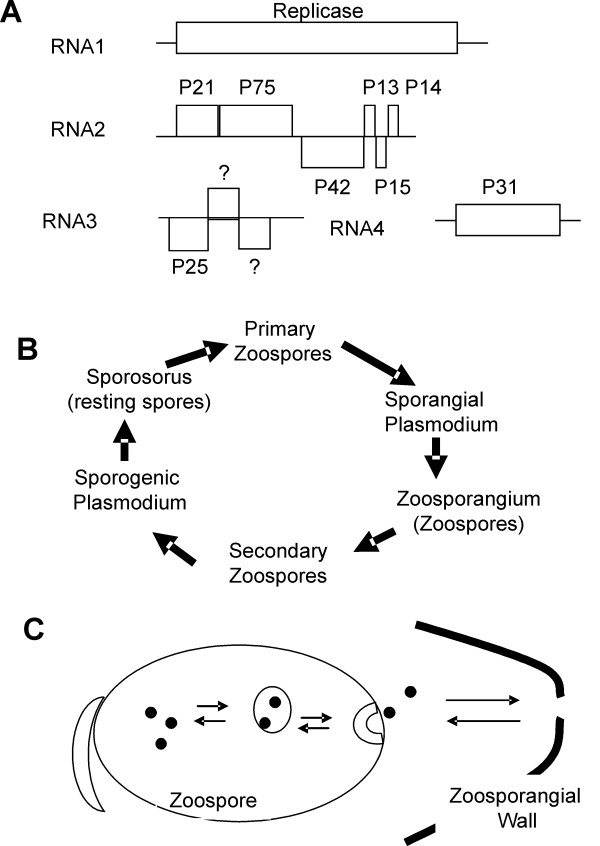
(**A**) Diagrammatic representation of the BNYVV genomes. Lines represent four genomic segments. Boxes represent coding regions. RNA2 is multicistronic. The 3' ORFs (P42, P13, P15, P14) are expressed from subgenomic RNAs. The names for each coding sequence are provided above the boxes. (**B**) Schematic of the *P. betae *life cycle. This shows the most significant developmental stages relating to this study. (**C**) Depiction of two models for virus transfer between plant cells and zoospores. Zoospore contains virus (black spheres) in the cytoplasm. Virus is transferred into vesicles (grey sphere, hemisphere), released to the exterior of the zoospore and then move into the plant cell through a break in the zoosporangial wall. These vesicles may be centers for virus replication or may be transport vesicles containing movement complexes or virions. In reverse, virus is acquired from an infected plant cell through a break in the zoosporangial wall. Virus is taken into the zoospore by pinocytosis. Particles may disassemble and be released into the zoospore cytoplasm for translation and replication.

The developmental cycle of *Polymyxa spp*. has two phases known as the sporangial and sporogenic stages [4-6] (Figure 1B). For *Polymyxa spp*, infection begins with penetration of the plant cell wall by swimming zoospores (Figure 1B). Zoospores transfer their cytoplasm into the plant cell and a multinucleate sporangial plasmodium develops. This matures into a zoosporangium containing numerous secondary zoospores. Mature zoosporangia have several lobes divided by cross walls. Exit tubes are generated from the zoosporangium into the plant extracellular space. Secondary zoospores are released into plant extracellular spaces through exit tubes extending from the zoosporangium [4-6]. These secondary zoospores penetrate new cells and sporogenic plasmodia develop. These mature into sporosori containing numerous resting spores [2,5-10]. Thick walled resting spores often remain in root debris in the soil after harvest. With a heavy rain or irrigation, resting spores germinate releasing primary zoospores to infect new roots or new plants and begin new rounds of infection [3].

Campbell first suggested that plasmodiophorids acquire virus when zoospore cytoplasm is injected into virus-infected plant cells [2]. The zoospore and plant cell cytoplasms have the opportunity to mix before membranes are laid down to form the sporangial plasmodium. According to this explanation, virus acquisition is accidental rather than an active transport mechanism. As plasmodiophorid development continues, virus accumulates inside resting spores waiting to be released with primary zoospores into the soil [3].

A recent study of *Soilborne wheat mosaic virus *(SBWMV) provided evidence of viral movement protein and RNA inside *P. graminis *resting spores [11]. SBWMV coat protein was absent. It was proposed that transfer of SBWMV into plant cells might require the viral movement protein to bind viral RNA and carry it through zoosporangial exit tubes into neighboring plant cells. This ribonucleoprotein complex may be similar to the movement complex which transports viral genomes between neighboring plant cells [11]. Moreover, evidence that SBWMV exists as a disassembled virus inside its vector led to speculations that the virus might also replicate inside its vector.

This study explores the relationship of *Beet necrotic yellow vein virus *(BNYVV) with its plasmodiophorid vector, *P. betae *[3]. BNYVV is the type member of the genus *Benyvirus*. BNYVV is a positive strand RNA virus with four genome segments (Figure 1A). Here we found all BNYVV proteins internally associated with *P. betae *resting spores and zoospores. Virions were not detected inside *P. betae *resting spores, lending further support to the notion that primary zoospores might not transmit encapsidated particles into their plant host. Evidence of viral replicase and other proteins inside fungal zoospores and resting spores suggests that BNYVV resides inside its vector throughout its developmental cycle.

## Results

### Immunodetection of BNYVV proteins in P. betae sporosori

**Figure 2:**
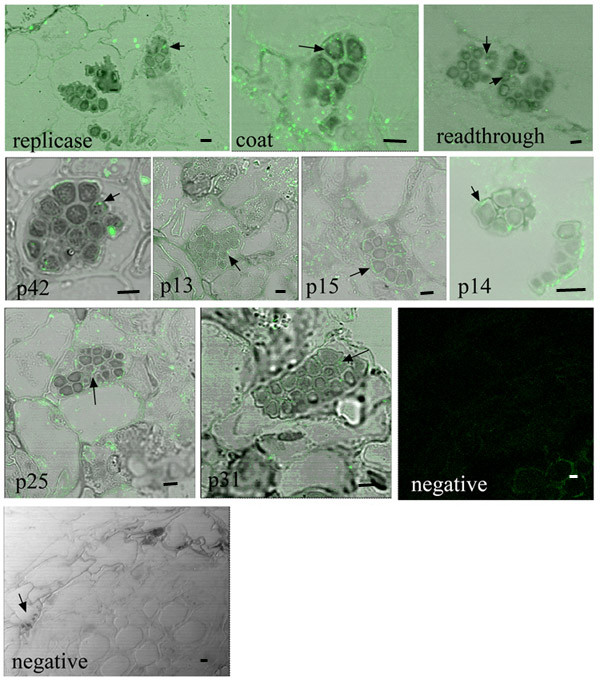
Examples of root cross sections which contain resting spores and were treated with each BNYVV antisera and FITC conjugated secondary antisera. The name of each viral protein is indicated in the bottom of each panel. The arrows point to examples of resting spores. Images were taken using confocal microscopy. Fluorescent images were merged with images taken using the transmitted light detector. Negative samples shown here were treated with buffer and secondary antisera. No label was detected in samples treated with buffer, BMV, or BSBMV antisera followed by FITC conjugated secondary antisera. Bars represent 10 μm.

Samples were initially screened using immunofluorescence labeling and confocal microscopy to detect BNYVV proteins associated with *P. betae *sporosori (Figure 2, Table 1). The thick walled resting spores were easy to view using the transmitted light detector of the confocal microscope. Drs. S. Bouzoubaa and D. Gilmer (IBMP, Strasbourg, France) provided antisera to each of the nine BNYVV proteins: replicase, coat, readthrough domain of the coat (RTD), P42, P13, P15, P14, P25, and P31 proteins [12]. These antisera have been well characterized and used for immunodetection of BNYVV proteins in infected plants [13-17]. Between 10 and 70 sporosori were treated with each antisera and a majority of resting spores tested positive for each BNYVV protein (Table 1). Nonspecific labeling was minimal in sections treated with buffer, BSMV, or BMV antisera (Table 1).

**Table 1:** Immunofluorescence labeling of *P. betae *resting spores

Antisera	Proportion cells with labeled sporosori
Replicase	30/30
CP	15/70
RTD	15/25
P42	25/25
P13	25/25
P15	10/10
P14	35/35
P31	15/15
P25	30/30
BMV^a^	0/5
No primary	0/40
BSBMV^a^	0/40

^a ^Antisera against two heterologous plant viruses produced negative results in all samples tested, indicating labeling was specific for BNYVV antigens.

**Figure 3:**
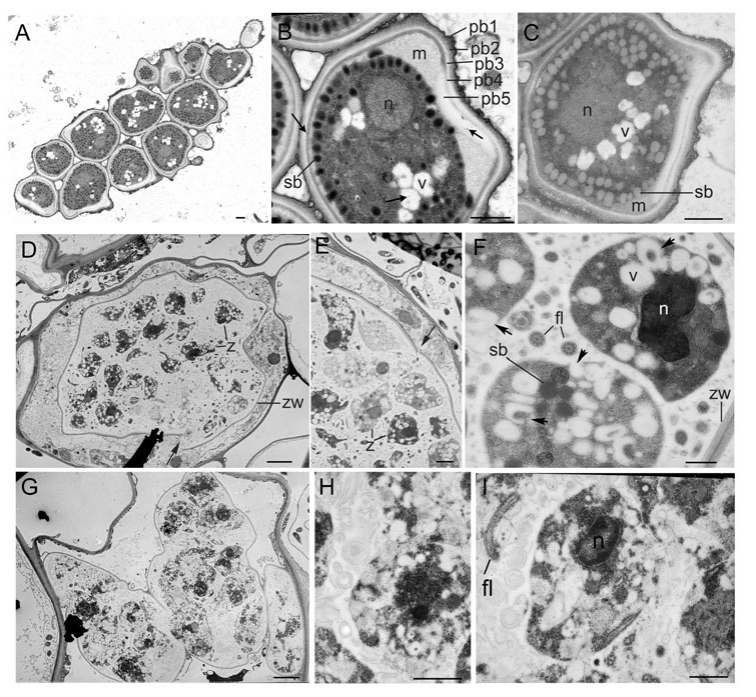
Transmission electron micrographs of *P. betae *sporosorus, resting spores, zoosporangia, and zoospores. (**A**) Micrograph shows sporosorus contains a cluster of 14 resting spores. (**B, C**) Micrographs show resting spores with different staining intensities. Arrows in b point to gold particles detecting viral replicase. Pb1, Pb2, Pb3, Pb4, Pb5 indicate the layers of the cell wall. The nucleus (n), storage bodies (sb), and matrix (m) are indicated. c, sample treated with buffer shows no gold particles. (**D, E, F**) Zoosporangium and zoospores (z) from virus infected plants. (**D, E**) Arrows point to gaps in the zoosporangial wall (zw). Individual zoospores (z) are identified. (**F**) Micrograph of zoospores show the nucleus (n), storage bodies (sb), vacuoles/vesicles (v), flagella (fl). Arrows point to various irregularly shaped vesicles. Some seem to extrude into the exterior of the zoospore. (**G, H, I**) Zoospornagium and zoospores in virus-free plants. In comparing (F) and (H), the healthy zoospores are highly vacuolated. Bars in **A, B, C, F, H, I**, represent 1 μm. Bars in **D, E, G**, represent 10 μm.

Further experiments were conducted using immunogold labeling and transmission electron microscopy where we could study morphological features of *P. betae *sporosori, resting spores, zoosporangia, and zoospores (Figure 3). *P. betae *resting spores had thick walls with five successive layers, labeled Pb1-Pb5 [10] (Figure 3A and 3B). Pb5 is an electron opaque zone, that is also known as the plug or apical cap in *P. graminis*, and may play a role in spore germination [8-10](Figure 3B and 3C). The lightly stained layer below Pb5 we named the matrix but is also called the fimbrillar matrix [8,10]. The matrix is often is seen as one or more conical projections extending beyond the Pb5 layer (Figure 3B and 3C). The darker gray spherical body containing the nucleus, vacuoles, and storage bodies is termed the "central body" for purposes of this study. Storage bodies are dispersed throughout the central body and vary in their staining intensity from grey in immature resting spores to nearly black in mature resting spores [8](Figure 3B and 3C). Mature resting spores also contain mitochondria, strands of ER, and vacuoles [9].

Thin sections were treated with antiserum detecting BNYVV replicase, coat, RTD, P42, P13, P15, P14, P25, and P31 proteins (Table 2). The presence of immunogold label in the spore wall (Pb1-4), Pb5, matrix, storage bodies, vacuole, central body of the spore, and in areas between spores was quantified. The average numbers of gold particles in each location was calculated in 1 μm^2 ^fields (Table 2). Control samples were treated with buffer and secondary antiserum (Figure 3C and 3F). In general, all antisera labeled resting spores and areas between resting spores (Table 2). Statistical analysis was used to test whether any of the viral proteins localized preferentially to a specific structure. Pairwise comparisons of values obtained for resting spores treated with each antiserum and buffer were conducted. Table 2 shows the replicase antiserum labeled most structures within the resting spores (Figure 3B, Table 2). Proteins that cooperate for specific functions in plants, such as encapsidation, replication, or cell-to-cell movement, do not have the same subcellular distribution in resting spores. For example, coat protein was greatest in the Pb5 layer while RTD occurs in storage bodies and the central region of the spore. The three movement proteins P42, P13, and P15 had different subcellular accumulation patterns [18]. P42 was dispersed throughout the resting spore. P13 was preferentially in the wall, matrix, and between spores. P15 was mainly in the storage body and central body of the spore. RTD, P13, and P15 are transmembrane proteins with different properties [19-21]. Perhaps both RTD and P15 associated with membranes of the storage body. Since we were unable to discern membraneous structures within the central body of the spore, matrix, or along the wall, we can not conclude whether these viral proteins were membrane associated. P14 is a cysteine rich protein [22] which, similar to P42, was dispersed through the resting spore. P25 and P31 are factors required for symptom development and plasmodiophorid transmission into plants [23]. The BNYVV P25 protein co-localized with replicase, RTD, and P15 in the storage bodies and central body of the spore. The P31 protein was mainly between spores.

**Table 2:** Distribution of immunogold label in virus infected *P. betae *resting spores^a^

Antisera	No. Fields (1 μm^2^)	Wall Pb1-4^b^	Pb5^b^	Matrix^c^	Storage Bodies	Vacuole	Central Body	Between Spores
Replicase	40	**6.52 ± 1.69**	**6.29 ± 1.23**	**6.90 ± 2.20**	**12.38 ± 3.32**	ND	**11.04 ± 2.45**	**3.45 ± 1.65**
Coat	35	1.16 ± 0.24	**4.92 ± 1.93**	1.53 ± 0.74	1.55 ± 0.49	2.46 ± 0.74	1.52 ± 0.36	1.90 ± 0.66
RTD	114	**2.76 ± 0.32**	2.12 ± 0.61	0.94 ± 0.36	**2.65 ± 0.57**	2.51 ± 0.40	2.14 ± 0.55	1.21 ± 0.28
P42	49	0.91 ± 0.15	0.88 ± 0.37	0.67 ± 0.36	1.32 ± 0.33	1.23 ± 0.48	1.08 ± 0.36	0.67 ± 0.25
P13	73	**4.55 ± 0.75**	2.12 ± 0.61	**2.31 ± 0.51**	1.98 ± 0.56	1.00 ± 0.39	1.61 ± 0.57	**3.05 ± 1.17**
P15	34	**3.48 ± 0.55**	1.27 ± 0.54	1.33 ± 0.50	**3.55 ± 0.68**	0.14 ± 0.14	3.67 ± 0.91	0.40 ± 0.31
P14	47	**2.23 ± 0.29**	2.70 ± 1.08	0.38 ± 0.16	0.43 ± 0.12	1.25 ± 0.52	0.80 ± 0.39	1.07 ± 0.22
P25	78	**4.48 ± 0.42**	3.07 ± 0.90	2.29 ± 0.49	**2.62 ± 0.53**	0.67 ± 0.33	**2.58 ± 0.55**	1.27 ± 0.39
P31	59	**4.28 ± 0.48**	2.43 ± 0.72	0.70 ± 0.22	0.68 ± 0.20	1.67 ± 0.66	1.42 ± 0.34	**3.71 ± 0.96**
Buffer	54	0.38 ± 0.22	0.00 ± 0.00	0.19 ± 0.10	0.40 ± 0.18	0.27 ± 0.23	0.10 ± 0.07	0.08 ± 0.08

^a ^All data were analyzed using PC SAS Version 9 (SAS Institute, Cary, NC). Analysis of variance (ANOVA) models were created to account for variability attributed to subcellular location and antisera for the total number of fields. Pairwise comparison of each antiserum treatment with buffer, for each location was carried out using ANOVA methods. Means and standard errors are reported. Values in bold identify means that differ significantly from the buffer control at p < 0.05.

^b ^*P. betae *resting spores have 5 wall layers (Pb1-5). Data for Pb5 was scored separately from Pb1-4. This is because Pb5 is thicker and distinct from layers Pb1-4. It is also suggested to have a role in spore germination, making it functionally distinct from the other wall layers. Because Pb5 might be functionally distinct from the other wall layers we decided to score Pb5 separately from Pb1-4.

^c ^The matrix is the cytoplasmic layer between the wall and the central body of the resting spore. It stains light grey and often forms conical projections extending beyond Pb5. Its function is unknown.

### Immunodetection of BNYVV proteins in P. betae zoosporangia

The *P. betae *zoosporangium is divided into lobes [6,8](Figure 3D and 3G). Zoosporangial cross walls retain zoospores in the separate compartments. At maturity, the cross walls break down and zoospores move across the compartments for release through the exit tubes (Figure 3G). There were places where the outer zoosporangial wall is in contact with the plant cell wall [6,8,24](Figure 3G). Figures 3D and 3E contain examples where there was a gap in the zoosporangial wall. The gap might be a break due to mechanical damage to the wall in intact plant cells. It is worth speculating that mechanical damage to the zoosporangial wall might create a site for exchange of virus between zoosporangia and plant cells.

Virus infected and virus-free root samples containing *P. betae *zoosporangia were compared (Table 3). While the virus infected samples and healthy samples were grown in infested soil for the same period of time, we were unable to identify resting spores in roots containing healthy *P. betae*. Although, virus-infected and virus-free (healthy) roots were similar in age, the zoosporangia were at slightly different developmental stages (Figure 3G, H, and 3I). Most likely, the roots were not simultaneously infected with *P. betae*, making it difficult to identify samples of a similar age.

**Table 3:** Distribution of immunogold label in virus infected and healthy *P. betae *zoospores

Antisera	*P. betae *Samples^a^	No. Fields (1 μm^2^)	Cytoplasm^b^	Storage Body	Vacuoles/Vesicles^c^	Nucleus	Flagella	Between Zoospores^d^	Zoosporangial Wall
Replicase	Infected	50	**7.35 ± 1.68o**	**2.40 ± 0.67o**	**6.19 ± 1.21o**	1.33 ± 0.33	1.56 ± 1.18	**4.11 ± 1.60o**	**9.04 ± 2.14o**
	Healthy	7	**0.00 ± 0.00**	**0.00 ± 0.00**	**0.00 ± 0.00**	ND	0.00 ± 0.00	**0.20 ± 0.20**	**0.00 ± 0.00**
Coat	Infected	98	1.17+0.22	0.21+0.08	2.57+0.42**o**	0.73+0.37	0.32+0.19	1.00+0.25	1.00+0.77
	Healthy	ND	ND	ND	ND	ND	ND	ND	ND
RTD	Infected	22	2.14 ± 0.59**o**	0.24 ± 0.18	3.10 ± 0.97**o**	1.00 ± 0.58	0.54 ± 1.21	0.50 ± 0.32	ND
	Healthy	ND	ND	ND	ND	ND	ND	ND	ND
P42	Infected	95	**5.30 ± 1.51o**	**1.32 ± 0.33o**	**5.03 ± 0.95o**	0.81 ± 0.29	0.19 ± 0.11	**7.05 ± 0.77o**	1.00 ± 0.71
	Healthy	18	**0.07 ± 0.26**	**0.00 ± 0.00**	**0.40 ± 0.74**	0.00 ± 0.52	0.33 ± 0.00	**0.50 ± 0.82**	0.60 ± 1.34
P13	Infected	111	4.38 ± 0.81**o**	0.60 ± 0.28	**2.38 ± 0.67o**	0.44 ± 0.29	**1.38 ± 0.50**	**2.13 ± 0.39**	2.33 ± 0.80
	Healthy	64	0.00 ± 1.08	0.07 ± 0.34	**0.39 ± 0.89**	0.53 ± 0.80	**0.00 ± 0.00**	**0.81 ± 1.27**	0.00 ± 0.00
P15	Infected	34	0.22 ± 0.10	0.40 ± 0.20	0.70 ± 0.21	1.67 ± 0.54	1.00 ± 0.44	0.71 ± 0.29	0.63 ± 0.22
	Healthy	24	0.26 ± 0.14	0.55 ± 0.15	1.24 ± 0.38	0.00 ± 0.00	0.18 ± 0.18	0.00 ± 0.25	0.75 ± 0.00
P14	Infected	119	1.29 ± 0.19	0.46 ± 0.10	0.98 ± 0.17	0.75 ± 0.37	0.27 ± 0.08	1.28 ± 0.17	1.48 ± 0.38
	Healthy	60	**0.05 ± 0.03**	0.06 ± 0.03	0.49 ± 0.12	0.00 ± 0.00	0.00 ± 0.00	0.58 ± 0.14	0.22 ± 0.15
P25	Infected	105	**2.83 ± 0.33o**	**1.55 ± 0.34o**	2.92 ± 0.39**o**	**2.88 ± 1.17o**	**0.91 ± 0.39**	1.93 ± 0.34	1.67 ± 1.31**o**
	Healthy	9	0.10 ± 0.10	**0.00 ± 0.00**	0.80 ± 0.44	**0.00 ± 0.00**	**0.00 ± 0.00**	ND	0.60 ± 0.24
P31	Infected	56	**3.48+0.59o**	**1.30+0.31o**	**4.10+0.54o**	**3.40+1.68o**	**3.38+0.77o**	**3.96+0.44o**	**4.00+1.14o**
	Healthy	75	**0.44 ± 0.10**	**0.08 ± 0.05**	**0.26 ± 0.08**	**0.27 ± 0.13**	**0.40 ± 0.31**	**0.91 ± 0.12**	**0.09 ± 0.09**
Buffer	Infected	110	0.27 ± 0.14	0.02 ± 0.02	0.38 ± 0.13	0.13 ± 0.10	0.10 ± 0.06	0.85 ± 0.18	0.13 ± 0.13
	Healthy	75	0.17 ± 0.10	0.00 ± 0.00	0.09 ± 0.07	0.17 ± 0.08	0.00 ± 0.00	0.00 ± 0.08	0.22 ± 0.00

^a ^Pairwise comparisons of each antiserum treatment with buffer or healthy samples, for each location was carried out using ANOVA methods as in Table 1. Means and standard errors are reported. Values with a **o **symbol identify means that differ significantly from the buffer control at p < 0.05. Values indicated in bold are virus infected and virus-free (healthy) samples that differ significantly, p < 0.05.

^b ^*P. betae *zoosporangia were identified in virus infected and healthy plants. These were isolated and compared for their reactivity with the various BNYVV antiserum.

^c ^Cytoplasm refers to the body of the zoospores.

^d ^Vacuoles and pinocytotic vesicles are grouped together. Pinocytotic vesicles seemed to form horseshoe or doughnut shaped bodies surrounding zoospore cytoplasm. Vacuoles are round or irregularly shaped vesicles that have no contents or fibrillar material.

**Figure 4:**
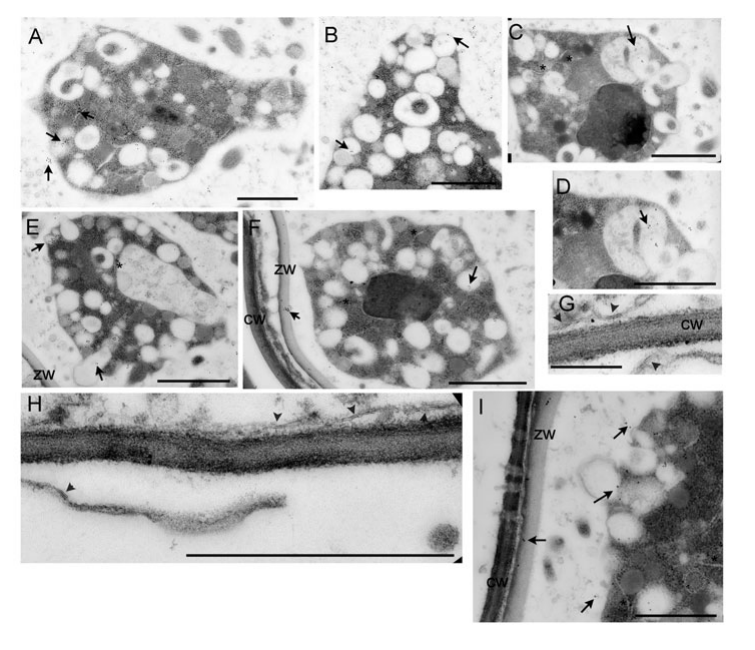
Immmunogold label of virus infected *P. betae *zoospores. (**A, B**) Arrows point to gold particles detecting viral replicase. Note in a, the arrows are in a row pointing to gold particles in the cytoplasm, vesicles, and extracellular space. This could represent the path of virus movement between the zoospore and zoosporangial sac detailed in the model in Fig 1c. Vesicles are irregularly shaped. Some are horseshoe shaped, others are surrounding pockets of cytoplasm, and some are reaching outside of the zoospore body. (**C, D**) shows zoospore and vesicles treated with coat protein antiserum. Vesicles contain fibers of unknown origin. (**E, F**) Zoospores treated with P14 and P31 antiserum, respectively. Both label the zoospore vacuoles/vesicles and zoosporangial wall. (**G, H**) shows BNYVV virions (arrowheads) in plant cells, along the plant cell wall. Gold particle in both panels label coat protein or virions. Virions were not found inside zoosporangium or sporosorus. (**I**) shows zoosporangial wall (zw) near plant cell wall (cw) and plasmodesmata. ZW seems to block plasmodesmata. Gold particles label P31 proteins. There are unknown projections through plasmodesmata. Edge of zoospore shows vesicles extending into the zoosporangial milieu. Bars represent 1 μm.

Zoospores in the virus infected samples were mature condensed structures, amoeboid in shape, and uni-nucleate. Zoospores in the healthy samples were slightly younger as evidenced by their ovoid shape. The healthy zoospores were also uni-nucleate and contained few storage bodies (Figure 3F, H and 3I). Areas between zoospores were filled with vesicles and membranes which remained after division of the contents of the zoosporangia to produce zoospores (Figure 3F, H, and 3I) [24,25]. The flagella were obvious in the spaces between zoospores [24](Figure 3F and 3I). Healthy and virus infected zoospores were filled with numerous vacuoles and vesicles. Some were round bodies while others were irregular in shape. Some vesicles were horseshoe structures encircle or capture areas of cytoplasm (Figure 4A–F). Other vesicles resembled secretory or pinocytotic vesicles leading to the exterior of the zoospore (Figure 3F; Figure 4A–F) [24].

Immunogold labeling was conducted as for the samples containing *P. betae *resting spore. Pairwise comparisons of values obtained following treatment with each antiserum or buffer were conducted. Comparisons of virus infected and virus-free (healthy) samples were also conducted. The replicase and P42 antisera labeled the zoospore cytoplasm, storage bodies, vacuoles, and between zoospores at levels that were significantly different from buffer treated samples and healthy samples (Table 3, Figure 4A and 4B). The highest concentration of coat protein and RTD is in the vacuoles/vesicles (Table 3, Figure 4C and 4D). RTD was also prevalent in the zoospore cytoplasm. P13 was prevalent in the cytoplasm and vacuoles/vesicles. P15 and P14 were scattered, and did not localize to one specific site (Figure 4E). Significant levels of P25 and P31 proteins, which are required for transmission and virus accumulation in roots, were detected in most locations (Figure 4F and 4I). Interestingly, P25 was found in the nucleus, as has been reported in plant cells (Table 3). One of the most obvious outcomes of the data presented in Table 3 is that significant levels of most BNYVV proteins were found in the cytoplasm and inside vacuoles/vesicles (Table 3). In Figure 4A, gold particles label the replicase in neighboring cytoplasm, vesicles, and zoospore exterior. It is worth speculating that the proteins were translated along the ER or free ribosomes in the zoospore cytoplasm. Replication complexes or virions may be packaged into vesicles and transported out of the zoospore. Alternatively, virus may be acquired from the exterior into vesicles, disassembled and then RNA is released into the cytoplasm for translation, replication (Figure 2C).

Investigations were conducted to identify virions, hoping to gain clues as to how virus is transferred between *P. betae *and root cells. Virions were reported previously to be associated with the outer membrane of the plasmodium, but not with internal structures [25,26]. Here we detected virus particles along the plant cell wall in root cells containing zoosporangia and in other root cells. These also were positive for immunolabelling with coat protein antiserum (Figure 4G and 4H). In previous studies of embedded zoospores, it was suggested that fibers inside the vacuoles/vesicles were virus particles [26](Figure 4D). However, since the fibers observed in this study did not reflect the dimensions of BNYVV particles, they may represent intermediate or disassembled structures. There were channels throughout the zoospore cytoplasm. Even at highest magnifications, we could not discern whether these were true channels or negatively stained virions (Figure 4A, C, D, E, and 4I). Since immunolabelling with coat protein antiserum did not preferentially label the channels, we have no evidence to indicate they were viral in origin (Figure 4C, D, and 4E).

## Discussion

This study tried to address two issues: 1) what is the nature of BNYVV association with its vector; and 2) how is virus transferred between the vector and root cells. Using immunofluorescence and immunogold labeling techniques, all BNYVV proteins were detected in *P. betae *resting spores and zoospores. Samples were also treated with buffer or heterologous antiserum (Figure 2) and these produced negative results, indicating that the immunolabelling was specific for BNYVV proteins. Comparing the immunogold label in BNYVV and healthy *P. betae *zoosporangia, provides further evidence that each BNYVV protein occurs inside virus containing *P. betae *zoosporangia.

There are two possible explanations for detection of all BNYVV proteins inside *P. betae *sporosori and zoosporangia. The first model, proposed by Campbell, suggests that the *Polymyxa *and plant cytoplasms have opportunities to mix and that virus may be freely exchanged on these occasions. Campbell proposed that this may occur before membranes are laid down to form the sporangial plasmodium [2]. The data presented in this paper support this model. Furthermore, we found occasional breaks in the zoosporangial wall which could create additional opportunities for the *P. betae *and plant cell cytoplasms to mix. If BNYVV is replicating in the same plant cell that is infected with *P. betae*, then it is reasonable to consider that all BNYVV proteins move freely between *P. betae *when there are breaks in the zoosporangial wall as well as during developmental of the sporangial plasmodium.

An alternative explanation is that *P. betae *is a host as well as a vector for BNYVV. The presence of viral replicase inside *P betae *resting spores and zoospores may be evidence that BNYVV replicates inside its vector. According to this model, accumulation of viral replicase, coat protein, RTD, P25, and P31 proteins, which are expressed from the genomic RNAs, suggests that viral RNAs may be translated as *P. betae *progresses through its life cycle. The P42, P13, P15, and P14 proteins are produced from subgenomic RNAs derived from BNYVV RNA2. Subgenomic RNA expression is dependent on production of minus strand RNAs involved in virus replication.

Considering the *P. betae *life cycle (Figure 1B), it is reasonable to consider that the spread of BNYVV within the developing zoosporangial thallus into the innumerable secondary zoospores, requires the virus to multiply within its vector. Similarly, following penetration of secondary zoospores into plant cells, the developing plasmodium may take up virus from the plant cell cytoplasm. However, virus multiplication is likely for BNYVV to spread within the developing sporosorus and most of the resting spores. Immunolabelling of sporosori and zoosporangia, which represent the sporogenic and sporangial stages of the *P. betae *life cycle, showed that each BNYVV protein associates with both life cycle stages. This could be evidence of different cytoplasms mixing or evidence that BNYVV multiplies inside its vector.

Tables 1 and 2 compare the subcellular distribution of the BNYVV proteins to determine if they localize to specific regions of the spores. In resting spores, the concentration of viral replicase was greatest in the cell wall, but was significant in most structures. Each of the BNYVV proteins had distinct subcellular accumulation patterns in resting spores, making it difficult to identify specific centers for viral activities. In zoospores, most of the BNYVV proteins are greatest in the cytoplasm, vesicles, and areas between spores. If BNYVV replicates inside *P. betae *zoospores, then these data could be explained by two different models (Figure 1C). Possibly, the BNYVV proteins are translated on free ribosomes in the cytoplasm, and are captured into secretory vesicles for virus replication and packaging. Virions, viral replication complexes, or viral movement complexes are then carried to the exterior of the cell. Alternatively the flow of events is in the other direction: virus may be captured from the cell exterior by pinocytotic vesicles. Virions may disassemble in the vesicles and viral RNA is released in the cytoplasm for translation and replication (Figure 3G and 4A).

The subcellular accumulation patterns for BNYVV P25 and P31 were intriguing because these proteins are suggested to play significant roles in virus transmission and accumulation in roots [23,27]. Here we show that both proteins accumulate to significant levels in *P. betae*. In this study P25 and P31 are the only proteins that localize to the zoospore nucleus. Prior studies in plant cells also show P25 traffics to the nucleus [13]. If P25 and P31 are actively transported into the nucleus, this would suggest that they are actively interacting with cellular components and that they may be functional within the P. betae zoospore. It has been suggested that nuclear accumulation of P25 may play a role in symptom expression in plants [13]. It would be intriguing to learn if P25 has similar abilities to cause disease symptoms in *P. betae*.

Further investigations are needed to determine if *P. betae *is a host for BNYVV. This requires developing research tools to study the time course of viral RNA accumulation in *P. betae *sporosori and zoosporangia. While there are examples of plant RNA viruses which multiply inside their insect vectors, and plant viruses that can replicate in yeast, there are no examples yet of plant viruses replicating inside plasmodiophorid vectors. BNYVV requires the RTD to mediate vector transmission to plants. This study revealed that there is an opportunity for more than one BNYVV protein to participate in vector acquisition and transmission. As we develop more tools for studying the association of BNYVV viruses with its vector, we will learn if transmission is actively enabled by specific viral proteins, or is the result of passive mixing of two cytoplasms as suggested by Campbell [2].

## Methods

### Plant materials

Seed of sugar beet cultivar Hilleshog 9155 were planted in the greenhouse in a field soil naturally infested with viruliferous *P. betae*. Bait plants were grown for about 12 weeks, with ambient temperature ranging from 16 – 30°C, irrigated as needed, and then harvested. Roots were washed free of soil and tested by DAS-ELISA for presence or absence of BNYVV.

### LR-white embedding

Root systems that tested positive for BNYVV, which necessitated infection with *P. betae*, were fixed for 2 h under vacuum in a solution containing 0.5% glutaraldehyde, 4% paraformaldehyde and 100 mM sucrose in 50 mM sodium cacodylate buffer (pH 7.2). Fixed roots were screened using light microscopy to identify segments containing *P. betae *zoosporangia or sporosori. These segments were embedded in LR-White, sectioned, and stained with a mixture of uranyl acetate and lead citrate, as described previously [11]. There were between one and five *P. betae *infected root cell in each root cross section analyzed.

### Immunolabelling

Drs. S. Bouzoubaa and D. Gilmer (IBMP, Strasbourg, France) provided antisera to each of the nine BNYVV proteins: replicase, coat, readthrough domain of the coat (RTD), P42, P13, P15, P14, P25, and P31 proteins[12]. Each antiserum was cross-reacted with dried plant extracts to eliminate nonspecific labeling of infected samples. Cross reacting each antiserum with plant extracts provided a control, demonstrating specificity of the immunolabelling reactions. BMV and BSBMV antisera were prepared in our laboratories and used in prior studies (Verchot et al., 2001).

Thick sections (1 μm) and ultrathin sections (60 nm) were cut using a diamond knife on a Sorvall MT 6000 ultramicrotome. Thick sections were affixed on ProbeOn Plus slides (Fisher Biotechnology, Pittsburgh, PA, USA) and ultrathin sections were mounted on formvar coated nickel grids (Electron Microscopy Science Inc., Hatfield, PA, USA). Immunofluorescence labeling of thick sections was conducted as described previously (Driskel et al., 2004, Verchot et al., 2001). A Leica TCS SP2 (Leica Microsystems, Bannockburn, IL, USA) confocal imaging system was used to study FITC-labeled root cross sections.

Immunogold labeling of ultrathin sections was conducted using each antiserum as described previously [28]. Thin sections were incubated in blocking solution consisting of phosphate buffer saline pH 7.5 (PBS; 130 mM NaCl, 7.0 mM Na_2_HPO_4_, 3.0 mM NaH_2_PO_4_) plus 2% bovine serum albumin (BSA; w/v) for 15 min, and then incubated with 2% normal goat serum in PBS plus 2% BSA for 15 min. Then samples were incubated with primary antisera diluted 1:500 in PBS plus 2% BSA (w/v), or buffer containing no primary antisera for 2 h. The grids were then washed five times for 5 min with PBS and then with PBS plus 2% fish gelatin (v/v) for 15 min. The grids were then incubated for 1 h with 10 nm gold conjugated rabbit antisera (EY Labs Inc. San Mateo, CA, USA) diluted 1:10 in PBS plus 2% fish gelatin. Grids were washed three times for 5 min with ddH_2_O, and stained with a solution of 2.5% uranyl acetate and 70% methanol (v/v) for 30 min, and then with a solution of 2% Reynold's lead citrate pH 12.0 (in ddH_2_O) for 20 min. Samples were washed with lukewarm ddH_2_O three times for 5 min and then dried. Control samples were incubated with blocking solution plus 10 nm gold-conjugated rabbit antisera (EY Laboratories). The distribution of gold particles in resting spores and zoosporangia were recorded in Tables 2 and 3.

### Confocal and transmission electron microscopy

Fluorescent samples were studied using a Leica TCS SP2 (Leica Microsystems, Bannockburn, IL, USA) confocal imaging system. The Leica TCS SP2 system was attached to a Leica DMRE microscope. The microscope was equipped with epifluorescence and water immersion objectives. For confocal microscopy, a krypton/argon laser was used to examine fluorescence. A 488-nm excitation wavelength was used to view FITC labeled samples. Electron microscopic analysis of samples was carried out using a JEOL JEM100 CXII scanning transmission electron microscope. Photographs were taken and developed in a dark room and then scanned using an HP scan jet 4570c. All images, obtained by confocal or electron microscopy, were processed using Adobe Photoshop CS version 8.0 software (Adobe Systems, San Jose, CA, USA).

## Competing interests

The author(s) declare that they have no competing interests.

## Authors' contributions

J. V-L conceived the study, carried out immunolabelling, microscopy, and wrote manuscript C. M. R. participated in the design of the study, grew and maintained sugar beets, prepared viruliferous and aviruliferous samples, aided with data interpretation and manuscript editing.

M. P. provided the statistical analysis of all data.

T. C. carried out LR-white embedding of root samples, performed all microtome sectioning, and developed all negatives.

All authors read and approved the final manuscript.
